# Extended Wearable ECG Monitoring Detects Unrecognised Arrhythmias After COPD Exacerbation: The PRECURSOR Pilot Study

**DOI:** 10.1111/crj.70240

**Published:** 2026-11-02

**Authors:** Ourania S. Kotsiou, Eudoxia Gogou, Dimitra Siachpazidou, Maria Pinaka, Fotini Bardaka, Antonios Garyfallos, Dimitrios Mantzaris, Alexandros Garyfallos, Athanasios Konstantinidis, Konstantinos I. Gourgoulianis

**Affiliations:** ^1^ Department of Respiratory Medicine University General Hospital of Larissa, University of Thessaly Larissa Greece; ^2^ Department of Physiology, Faculty of Medicine University of Thessaly Larissa Greece; ^3^ MOKAAL Information Technology Company Ioannina Greece; ^4^ Department of Nursing University of Thessaly Larissa Greece; ^5^ Department of Respiratory Medicine University Hospital of Ioannina, University of Ioannina Ioannina Greece

**Keywords:** ambulatory electrocardiography, arrhythmia, cardiovascular risk, COPD exacerbation, remote patient monitoring, wearable device

## Abstract

**Background:**

The period after hospitalisation for an acute exacerbation of chronic obstructive pulmonary disease (COPD) is associated with cardiovascular vulnerability. Clinically important rhythm disturbances may be intermittent or asymptomatic and escape routine postdischarge assessment; however, the diagnostic yield of prolonged monitoring is intrinsically influenced by monitoring duration.

**Methods:**

In this prospective, single‐centre observational pilot, 18 adults recently hospitalised for COPD exacerbation underwent continuous remote monitoring for 21 days between September 2019 and February 2020. The three‐lead ZENSOR wearable recorded electrocardiographic rhythm, heart rate and respiratory rate and transmitted full‐disclosure data and predefined alerts for clinician review. The primary outcome was participant‐level detection of previously unrecognised, cardiologist‐confirmed arrhythmias warranting therapeutic evaluation or intervention. Outcomes were analysed descriptively with exact 95% binomial confidence intervals.

**Results:**

Participants were predominantly male (15/18; 83.3%), with a mean age of 73 ± 6 years. Seven had GOLD grade 3–4 airflow limitation, seven received long‐term oxygen therapy, five had coronary artery disease and four had chronic atrial fibrillation. During the planned 21‐day monitoring period, previously unrecognized clinically actionable arrhythmias were detected in 9/18 participants (50.0%; 95% CI: 26.0–74.0). Nonmutually exclusive findings included trigeminy (2/18), third‐degree atrioventricular block (2/18), bradycardia (5/18), newly detected atrial fibrillation (1/18), supraventricular tachycardia (2/18) and nocturnal atrial or ventricular ectopy (8/18). Sixteen participants (88.9%; 95% CI: 65.3–98.6) reported satisfaction with monitoring.

**Conclusions:**

Extended wearable electrocardiographic monitoring produced a high participant‐level detection yield of previously unrecognised arrhythmias during early recovery from hospitalised COPD exacerbation. This finding supports targeted postdischarge rhythm surveillance but does not distinguish biological post‐exacerbation vulnerability from the greater ascertainment achieved by prolonged monitoring. Confirmation requires larger controlled studies with comprehensive cardiopulmonary phenotyping, standardised monitoring‐performance metrics and comparative clinical outcomes.

## Graphical abstract

In this prospective pilot, 21 days of continuous wearable ECG monitoring after hospitalisation for COPD exacerbation detected previously unrecognised, clinically actionable arrhythmias in nine of 18 participants (50%). These findings highlight a potentially important postdischarge surveillance gap and support further evaluation of targeted rhythm monitoring in high‐risk patients.

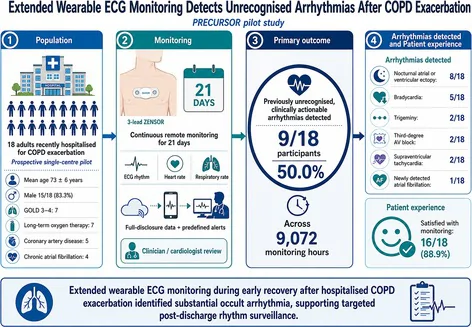

## 1 Introduction

Acute exacerbations of COPD are often approached primarily as respiratory events, although their biological and clinical consequences extend beyond the lungs. COPD and cardiovascular disease share major determinants, including ageing, cumulative tobacco exposure, systemic inflammation, autonomic dysfunction, hypoxaemia and physical deconditioning. During an exacerbation, additional perturbations—including increased work of breathing, adrenergic activation, acid–base and electrolyte disturbances and myocardial oxygen supply–demand imbalance—may further destabilise susceptible myocardium [1, 2, 3, 4]. Large contemporary cohorts consistently demonstrate that the risk of severe cardiovascular events and death increases promptly after moderate or severe COPD exacerbation and remains elevated beyond the acute episode [5, 6, 7, 8].

Cardiac arrhythmia is an important component of this excess cardiovascular risk. During hospitalised COPD exacerbations, pooled evidence indicates that rhythm disturbances are common and prognostically adverse, particularly when atrial fibrillation is present [9]. GOLD 2026 recommends electrocardiography at COPD diagnosis and when symptoms change unexpectedly and notes that Holter monitoring may be appropriate for intermittent dizziness or syncope because atrial fibrillation detection increases with longer monitoring duration [1]. The transition from hospital to home nevertheless creates a clinically important surveillance gap. A resting 12‐lead electrocardiogram samples only a brief interval, whereas conventional 24‐h Holter monitoring may fail to capture intermittent bradyarrhythmia, paroxysmal atrial fibrillation, supraventricular tachycardia or clinically relevant ectopy. In other at‐risk populations, extending ambulatory electrocardiographic recording beyond 24 h consistently increases diagnostic yield [10, 11, 12, 13, 14].

Wearable remote‐monitoring systems may bridge this postdischarge surveillance gap by combining prolonged physiological acquisition with near‐real‐time clinical review. Their clinical value, however, cannot be inferred from recording capacity alone: Signals must be interpretable, automated detections require expert confirmation and confirmed abnormalities must be linked to a prespecified response pathway. Recent continuous‐monitoring studies in COPD support the plausibility of clinically important occult arrhythmia while also underscoring substantial dependence on population selection and monitoring duration [15, 16]. Evidence specifically addressing continuous wearable electrocardiographic surveillance during the first weeks after COPD hospitalisation remains limited.

The PRECURSOR pilot evaluated a wearable mobile health platform during the early posthospitalisation period. The objectives were to estimate the 21‐day participant‐level detection yield of previously unrecognised arrhythmias judged to warrant therapeutic evaluation or intervention, characterise the observed rhythm abnormalities and assess patient‐reported satisfaction with the monitoring system.

## 2 Methods

### 2.1 Study Design and Participants

We conducted a prospective, single‐centre observational pilot at the Department of Respiratory Medicine, University General Hospital of Larissa, Greece, between September 2019 and February 2020. Adults with an established diagnosis of COPD were assessed following hospital treatment for an acute exacerbation. Eligible participants had completed treatment for the index exacerbation, were considered clinically stable for discharge and were able to use the remote‐monitoring equipment independently or with caregiver assistance. Exclusion criteria were haemodynamic instability, a continuing indication for inpatient cardiac or respiratory monitoring, inability to provide informed consent, cognitive or physical impairment that precluded device use in the absence of adequate caregiver support or technical and social circumstances that prevented reliable remote data transmission. Eighteen eligible patients were enrolled and completed the planned 21‐day study follow‐up period. The screening denominator, reasons for non‐enrolment and exact interval from hospital discharge to device placement were not retained and therefore cannot be reported. Pre‐existing stable atrial fibrillation was not an exclusion criterion because the study was designed as a pragmatic feasibility evaluation of postdischarge rhythm surveillance across a multimorbid COPD population rather than as an incidence study restricted to arrhythmia‐naïve participants. Accordingly, only newly detected atrial fibrillation was counted as a new AF finding, whereas other previously unrecognised rhythm disturbances could contribute to the primary outcome in participants with known AF.

The investigation was conducted in accordance with the Declaration of Helsinki and received prior approval from the Ethics Committee of the University General Hospital of Larissa. Before enrolment, each participant received oral and written information describing the study procedures, continuous physiological monitoring, remote data transmission, confidentiality protections, voluntary participation and the right to withdraw without consequences for clinical care. Written informed consent was obtained before any study‐specific procedure or device placement. Prospective clinical trial registration was not undertaken because this was a non‐interventional observational feasibility study.

### 2.2 Remote‐Monitoring System

Participants underwent continuous monitoring with the ZENSOR system (Intelesens Ltd), integrated within the PRECURSOR mobile health platform. Electrocardiographic rhythm, heart rate and respiratory rate were acquired continuously for a planned 24 h/day over 21 consecutive days. Data were relayed through the PRECURSOR wireless body‐area network and central information environment to a clinician‐facing event management point. The prespecified monitoring exposure was 504 h per participant and 9072 h across the cohort.

The ZENSOR system comprised a reusable, battery‐operated monitoring unit, a rechargeable battery pack and a single‐use self‐adhesive electrode array. Three conductive gel patch electrodes were connected to the monitoring unit through magnetic studs and positioned on the chest according to the manufacturer's instructions, enabling three‐lead electrocardiographic acquisition and impedance‐derived respiratory monitoring. ECG signals were sampled at 360 Hz with 12‐bit resolution and a bandwidth of 0.67–40 Hz; respiratory signals were sampled at 120 Hz with 12‐bit resolution, and a three‐axis accelerometer acquired activity data at 100 Hz. Full disclosure ECG, respiratory and accelerometry data were stored locally for subsequent review in the ZENSOR+ Ambulatory Full Disclosure ECG and Event Viewer. Predefined event alerts and patient‐activated recordings could also be transmitted through Wi‐Fi or a mobile hotspot to the ZENSOR online platform. On‐device algorithms screened for asystole, ventricular fibrillation, bradycardia, atrial fibrillation and tachycardia; automated detections were treated solely as screening alerts and required physician confirmation. Persistent trigeminy was not one of the listed automated screening categories; it was treated as a clinician‐adjudicated ECG rhythm finding identified on review of recorded ECG data. Recordings affected by noise, electrode detachment, signal loss or nonphysiological waveforms were excluded from diagnostic interpretation. Device specifications were cross‐checked against the US Food and Drug Administration 510(k) summary for the Intelesens ZENSOR system.

### 2.3 Arrhythmia Assessment and Clinical Action

The primary outcome was the proportion of participants in whom at least one previously unrecognized arrhythmia was confirmed during the 21‐day monitoring window and judged by the clinical team to warrant therapeutic evaluation or intervention. Clinically adjudicated rhythm abnormalities included persistent trigeminy, third‐degree atrioventricular block, bradycardia, newly detected atrial fibrillation and supraventricular tachycardia. Nocturnal atrial or ventricular premature beats were additionally recorded as a separate descriptive rhythm category. Rhythm findings were nonmutually exclusive.

Electrocardiographic recordings associated with automated or patient‐activated alerts were reviewed by cardiologists experienced in rhythm interpretation. ZENSOR+ session reports provided patient‐specific session dates and event timing for device‐detected alerts; automated output was not accepted as diagnostic in isolation. Each suspected episode required review of the corresponding ECG trace, with secondary cardiologist review when the initial interpretation was uncertain. Operational definitions included newly detected atrial fibrillation lasting ≥ 30 s; supraventricular tachycardia comprising ≥ 3 consecutive supraventricular beats at > 100 beats/min; bradycardia defined as a heart rate < 50 beats/min sustained for ≥ 30 s; persistent trigeminy defined as a repeating sequence of two sinus beats followed by a premature ventricular complex; and third‐degree atrioventricular block defined by complete atrioventricular dissociation. Nocturnal atrial and ventricular premature beats were recorded as a separate descriptive category.

Cardiologist‐confirmed clinically important abnormalities were communicated promptly to the responsible clinical team. Transmitted recordings and measurements were reviewed on an ongoing basis, with clinical review occurring within 48 h. Clinical action followed confirmation according to rhythm diagnosis, symptom burden, haemodynamic relevance, comorbidities and contemporaneous clinical guidance. Exact confirmation‐to‐treatment latency was not prospectively quantified; accordingly, the present analysis focuses on detection yield and feasibility rather than treatment‐effect estimates.

### 2.4 Baseline and Acceptability Measures

Baseline variables comprised age, sex, body mass index, educational level, caregiver availability, GOLD airflow‐limitation grade, long‐term oxygen therapy (LTOT), comorbidity count, coronary artery disease and pre‐existing chronic atrial fibrillation. Educational attainment was categorised as primary, secondary, tertiary or illiterate; the manuscript variable ‘low educational level’ corresponded to nontertiary education. Additional descriptive clinical information included hypercapnia/non‐invasive ventilation, pulmonary hypertension, cardiovascular medication, resting pulse and postdischarge azithromycin exposure where available. None of the participants received azithromycin during the 21‐day postdischarge monitoring period. Acceptability was assessed at completion of monitoring using a dichotomous satisfaction item. Device adherence was supported by confirmation that all 18 participants applied the wearable and generated ECG recordings stored and available for review within the ZENSOR+ software.

### 2.5 Statistical Analysis

Analyses were deliberately descriptive because this was a small, uncontrolled feasibility cohort. Continuous variables are reported as mean ± standard deviation and categorical variables as *n*/*N* (%). Exact two‐sided Clopper–Pearson 95% confidence intervals were calculated for the participant‐level detection yield and satisfaction proportion. The denominator was 18 unless explicitly stated otherwise. Rhythm categories were analysed as nonmutually exclusive outcomes.

## 3 Results

### 3.1 Participant Characteristics

All 18 enrolled participants completed the planned 21‐day study follow‐up period; 15 (83.3%) were men. Mean age was 73 ± 6 years and mean body mass index was 28 ± 4 kg/m^2^. Seventeen participants (94.4%) were classified as having a low educational level, defined as nontertiary education, and eight (44.4%) had an available caregiver. Seven (38.9%) had GOLD grade 3 or 4 airflow limitation, and seven were receiving LTOT. All had GOLD grade 3–4 airflow limitation, and four had recorded hypercapnia with BiPAP use, supporting the interpretation that oxygen‐treated participants represented a subgroup with more advanced respiratory disease (Table 1).

**TABLE 1:** Baseline characteristics.

Characteristic	Result
Participants	18
Age, years	73 ± 6
Male sex	15/18 (83.3%)
BMI, kg/m^2^	28 ± 4
Low educational level (nontertiary)^a^	17/18 (94.4%)
Caregiver available	8/18 (44.4%)
GOLD airflow‐limitation grade 3–4	7/18 (38.9%)
Long‐term oxygen therapy	7/18 (38.9%)
At least one comorbidity	17/18 (94.4%)
Number of comorbidities	3 ± 1
Coronary artery disease	5/18 (27.8%)
Known chronic atrial fibrillation	4/18 (22.2%)

*Note:* Values are mean ± SD or *n*/*N* (%).

Abbreviations: BMI, body mass index; GOLD, Global Initiative for Chronic Obstructive Lung Disease.

^a^ Low educational level was defined as nontertiary education.

Multimorbidity was common: 17 participants (94.4%) had at least one documented comorbidity, with a mean of 3 ± 1 conditions per participant. Coronary artery disease was present in 5/18 (27.8%), and 4/18 (22.2%) had known chronic atrial fibrillation at baseline. For all four participants with known AF, baseline resting pulse ranged from 62 to 84 beats/min; all four were receiving oral anticoagulation, three were receiving a beta‐blocker and one was receiving amiodarone. These observations provide no evidence of an uncontrolled rapid ventricular response at enrolment, although AF subtype (paroxysmal, persistent or permanent) was not available, and longitudinal ventricular‐rate control was not formally adjudicated. Pulmonary hypertension was explicitly documented in one participant in the available records; systematic echocardiographic assessment of pulmonary hypertension was not available.

### 3.2 Monitoring Exposure and Arrhythmia Detection

All 18 participants remained in the study throughout the planned 21‐day monitoring period and generated ECG recordings that were stored and available for review in ZENSOR+; quantitative device‐derived wear time and analysable signal duration were not available from the retained archive. The planned monitoring exposure was 504 h per participant over 21 days. At least one previously unrecognized arrhythmia judged to warrant therapeutic evaluation or intervention was detected in 9/18 participants, corresponding to a participant‐level yield of 50.0% (exact 95% CI: 26.0–74.0).

The nonmutually exclusive clinically adjudicated rhythm abnormalities comprised bradycardia in 5/18 participants (27.8%), persistent trigeminy in 2/18 (11.1%), third‐degree atrioventricular block in 2/18 (11.1%), newly detected atrial fibrillation in 1/18 (5.6%) and supraventricular tachycardia in 2/18 (11.1%). Nocturnal atrial or ventricular premature beats were additionally observed in 8/18 participants (44.4%) as a separate descriptive rhythm category (Table 2). Because individual participants could contribute to more than one clinically adjudicated rhythm category, category‐specific counts should not be summed to derive the participant‐level primary outcome.

**TABLE 2:** Arrhythmias detected during 21‐day continuous monitoring.

Monitoring outcome	*n*/*N* (%)	Exact 95% CI
Any previously unrecognized clinically actionable arrhythmia	9/18 (50.0%)	26.0–74.0
Bradycardia	5/18 (27.8%)	9.7–53.5
Persistent trigeminy	2/18 (11.1%)	1.4–34.7
Third‐degree atrioventricular block	2/18 (11.1%)	1.4–34.7
Newly detected atrial fibrillation	1/18 (5.6%)	0.1–27.3
Supraventricular tachycardia	2/18 (11.1%)	1.4–34.7
Nocturnal atrial or ventricular premature beats	8/18 (44.4%)	21.5–69.2

Rhythm categories were nonmutually exclusive; a single participant could contribute to more than one category, and category percentages should therefore not be summed. Confidence intervals are exact Clopper–Pearson intervals. Automated ZENSOR+ event labels were screening outputs and were not accepted as diagnoses in isolation; the reported categories reflect clinician‐reviewed ECG findings. Nocturnal atrial or ventricular premature beats were recorded as a descriptive category and did not independently define primary‐outcome status.

### 3.3 Patient Satisfaction

Sixteen of 18 participants reported satisfaction with the wearable monitoring system (88.9%; exact 95% CI: 65.3–98.6). All 18 participants applied the wearable and generated ECG recordings that were stored and available for review within the ZENSOR+ software, supporting feasibility of postdischarge ECG acquisition in this older, multimorbid cohort.

## 4 Discussion

In this prospective pilot, 21 days of continuous wearable monitoring identified at least one previously unrecognised arrhythmia judged to warrant therapeutic evaluation or intervention in half of the participants recovering from hospitalised COPD exacerbation. The observed spectrum included advanced conduction disease, bradycardia, supraventricular tachyarrhythmia, newly detected atrial fibrillation, trigeminy and frequent nocturnal ectopy. The central finding is therefore a high diagnostic yield during prolonged observation of a vulnerable recovery period—not evidence that the exacerbation itself caused a 50% increase in arrhythmia occurrence. The design cannot separate true post‐exacerbation electrophysiological vulnerability from the greater ascertainment expected when monitoring is extended from a brief ECG or 24‐h Holter to a planned 504 h per participant.

This signal is biologically and epidemiologically coherent. COPD exacerbations may intensify hypoxaemia, systemic inflammation, sympathetic activation, acid–base and electrolyte disturbances and myocardial oxygen supply–demand imbalance, whereas acute treatments and comorbid cardiovascular disease may further modify electrophysiological stability. Contemporary EXACOS‐CV analyses and related cohorts demonstrate that cardiovascular‐event risk increases sharply after COPD exacerbation and remains elevated for months [5, 6, 7, 8]. A 2025 meta‐analysis estimated a pooled arrhythmia prevalence of 15% during acute COPD exacerbations and found that arrhythmia—particularly atrial fibrillation—was associated with greater in‐hospital mortality [9]. Continuous implantable monitoring in 40 high‐risk COPD participants subsequently demonstrated that AF burden could cluster around exacerbations, including after hospitalised events [15]. PRECURSOR extends this clinical question to a distinct setting: wearable surveillance during the first 3 weeks of recovery at home.

The observed 50% participant‐level yield is higher than estimates derived from shorter or inpatient assessments, but it should not be interpreted as a directly comparable prevalence estimate. Detection probability is intrinsically dependent on monitoring duration, case mix, rhythm definitions, signal quality, alert configuration and adjudication intensity. Studies comparing extended patch monitoring with 24‐h Holter recording consistently show that additional monitoring time identifies events that would otherwise remain unobserved [10, 11, 12, 13]. This principle is explicitly acknowledged in GOLD 2026 [1]. Importantly, recent COPD‐specific evidence points in the same direction while illustrating why direct numerical comparisons are inappropriate: Coleman et al. reported actionable arrhythmias in 48% of 21 selected symptomatic patients with moderate‐to‐severe COPD during 12 months of implantable‐loop‐recorder monitoring, compared with 11% of matched controls [16], whereas Ding et al. found arrhythmias in 60.3% of 544 AECOPD hospitalisations monitored for at least 24 h but limited overall prognostic impact across arrhythmia subtypes [17]. The high yield in PRECURSOR may therefore reflect both genuine post‐exacerbation vulnerability and the substantially longer observation window; the present study was not designed to quantify the independent contribution of either mechanism.

Several abnormalities had potential management implications. Third‐degree atrioventricular block and clinically important bradycardia can justify urgent rhythm evaluation and, in selected cases, pacing assessment; newly detected atrial fibrillation can alter thromboembolic‐risk evaluation and anticoagulation decisions. In PRECURSOR, transmitted measurements were clinically reviewed within 48 h, clinically important abnormalities were recognised at review and confirmed findings were communicated promptly for clinical management. The study was not designed to determine whether monitoring accelerated treatment, prevented adverse events or changed readmission or survival; it therefore establishes detection feasibility and a workable clinical‐response pathway rather than therapeutic effectiveness.

Disease severity and comorbidity are central alternative explanations for the observed rhythm burden. Seven participants were receiving LTOT, and seven had GOLD grade 3–4 airflow limitation. Among seven LTOT recipients with detailed severity data, all had GOLD grade 3–4 airflow limitation, and four had recorded hypercapnia with BiPAP use, supporting the interpretation that oxygen‐treated participants represented a more advanced respiratory‐disease subgroup. The contribution of chronic hypoxaemia, ventilatory failure, pulmonary vascular disease or right‐heart strain cannot be isolated from the recent exacerbation. Pulmonary hypertension was documented in one participant, although formal echocardiographic pulmonary‐pressure and right‐heart indices were not available for systematic cohort‐wide phenotyping. Likewise, bradycardia and third‐degree atrioventricular block may reflect intrinsic conduction disease, ischaemic or structural heart disease, sleep‐disordered breathing, medication effects or combinations of cardiopulmonary factors rather than COPD per se.

The inclusion of four participants with known chronic atrial fibrillation should also be interpreted in this pragmatic context. Stable pre‐existing AF was not excluded because the feasibility question concerned whether prolonged postdischarge monitoring could identify previously unrecognised clinically important rhythm abnormalities in a real‐world multimorbid COPD population, not only incident AF. Their resting pulse rates at baseline ranged from 62 to 84 beats/min; all four were receiving oral anticoagulation, three were receiving a beta‐blocker and one was receiving amiodarone. These observations provide no evidence of an uncontrolled rapid ventricular response at enrolment. AF subtype was not available, and the study did not assign a single cardiac or pulmonary cause to pre‐existing AF; accordingly, mechanistic interpretation remains necessarily cautious.

Medication‐related proarrhythmia was considered explicitly. None of the participants received azithromycin during the 21‐day postdischarge monitoring period. Antibiotic exposure during the index hospitalisation was not systematically recorded, so previous in‐hospital macrolide exposure cannot be evaluated. No obstructive sleep apnoea diagnosis was documented in the available clinical records. Coronary or structural heart disease, sleep‐disordered breathing and rate‐ or rhythm‐modifying medication therefore remain plausible contributors to the observed arrhythmias.

Patient‐reported satisfaction was high despite advanced age, substantial multimorbidity and the fact that 17/18 participants were classified as having nontertiary education. Educational attainment may influence digital‐health literacy, device handling, symptom recognition, communication with clinicians and access to follow‐up care, whereas caregiver support may modify these relationships. Importantly, all 18 participants applied the wearable and generated stored ECG recordings in ZENSOR+, supporting the feasibility of ECG acquisition across this cohort. A single satisfaction item cannot fully characterise comfort, burden, digital accessibility or caregiver dependence; future evaluations should incorporate validated usability and health‐literacy measures alongside standardised technical‐performance metrics.

## 5 Clinical Implications

These findings support further evaluation of a risk‐enriched, rather than universal, postdischarge monitoring strategy. Candidate enrichment factors could include severe exacerbation, established cardiovascular disease, baseline conduction abnormality, persistent hypoxaemia, pulmonary hypertension or right‐heart dysfunction, electrolyte disturbance, elevated resting heart rate, sleep‐disordered breathing or otherwise unexplained postdischarge symptoms. This proposal is hypothesis‐generating and should not be interpreted as evidence that any of these factors caused the arrhythmias observed in PRECURSOR. Any implementation pathway should prespecify alert recipients, urgency categories, maximum review times, confirmatory testing, escalation procedures and documentation of the clinical action attributable to each confirmed event.

## 6 Strengths and Limitations

The principal strength of PRECURSOR is the prolonged, continuous observation of a clinically vulnerable period that is rarely captured in routine COPD follow‐up. Monitoring extended across a planned 9072 participant‐hours in an older cohort with substantial airflow limitation and multimorbidity, providing a pragmatic proof of concept for home‐based rhythm surveillance.

The study also has important limitations. Its small, single‐centre, uncontrolled cohort was predominantly male (15/18; 83.3%), limiting precision and generalisability. Although patient‐specific session dates and device‐alert timing were recoverable from ZENSOR+ reports, automated alerts represented screening outputs rather than diagnoses, and the clinical endpoint relied on physician‐reviewed ECG findings. The historical archive did not retain complete participant‐by‐event linkage between every timestamped automated alert, the corresponding ECG tracing and the final cardiologist‐adjudicated diagnosis across the entire cohort. Similarly, although the original study summary retained the aggregate participant‐level primary‐outcome count, complete individual‐level linkage between baseline covariates and final adjudicated outcome status was not recoverable for all participants, precluding a valid retrospective comparison according to arrhythmia status. Accordingly, rhythm‐category counts should be interpreted as nonmutually exclusive participant‐level findings rather than event‐incidence rates, and automated alert counts should not be considered equivalent to clinically confirmed arrhythmias. Clinical review occurred within 48 h, but exact confirmation‐to‐treatment latency was not prospectively quantified. Detailed echocardiographic phenotyping and complete medication data from the index hospitalisation were not systematically available. Finally, in the absence of a comparator group, the study cannot determine whether monitoring improved treatment timeliness, reduced cardiovascular events or readmissions or affected mortality.

## 7 Future Research

A definitive multicentre study should prospectively register a detailed protocol, prespecify rhythm definitions and alert thresholds and retain structured linkage between device‐event timing, ECG tracings, cardiologist adjudication, confirmed diagnosis and subsequent clinical action. Baseline phenotyping should include spirometric severity, arterial or transcutaneous oxygenation, long‐term oxygen‐therapy indication and dose, echocardiography with pulmonary‐pressure/right‐heart assessment, coronary and structural heart disease, obstructive sleep apnoea, AF subtype and rate control and complete medication exposure including macrolides and rate‐modifying drugs. Technical performance should be reported through standardised objective wear time, analysable signal time, missingness patterns, false‐alert burden and alert‐to‐review latency. A contemporaneous comparator and an adequately powered sample will be required to assess determinants of arrhythmia detection and determine whether greater detection translates into improved clinical outcomes.

## 8 Conclusions

In this small prospective cohort, extended wearable electrocardiographic monitoring during the first 3 weeks after hospitalisation for COPD exacerbation yielded previously unrecognised arrhythmias judged to warrant therapeutic evaluation or intervention in one‐half of participants. The finding is clinically important but preliminary and should be interpreted as diagnostic yield rather than evidence of COPD‐specific causation or a direct effect of exacerbation on arrhythmia incidence. PRECURSOR demonstrates the feasibility and potential diagnostic value of postdischarge rhythm surveillance; confirmation now requires larger controlled studies with comprehensive cardiopulmonary phenotyping, standardised technical‐performance reporting and comparative clinical outcomes.

## Author Contributions

Ourania S. Kotsiou and Konstantinos I. Gourgoulianis conceived the clinical study. The clinical and engineering authors contributed to study implementation, participant monitoring, data acquisition and clinical interpretation. The corresponding author drafted the manuscript, and all authors critically revised the work, approved the final version and accepted accountability for its integrity. The final CRediT allocation should be confirmed by all authors before submission.

## Funding

The research is conducted in the operating framework of the University of Thessaly Innovation, Technology Transfer Unit and Entrepreneurship Center ‘One Planet Thessaly’, under the ‘University of Thessaly Grants for publication support’ action and is funded by the Special Account of Research Grants of the University of Thessaly.

## Ethics Statement

The study was approved by the Ethics Committee of the University General Hospital of Larissa and was conducted in accordance with the Declaration of Helsinki. All participants provided written informed consent before enrolment and device placement.

## Conflicts of Interest

Antonios Garyfallos and Alexandros Garyfallos were affiliated with MOKAAL, the information technology company involved in developing and implementing the PRECURSOR platform. The remaining authors declare no competing interest.

## Data Availability

The participant‐level clinical and electrocardiographic data contain sensitive health information and are not publicly available. De‐identified data may be made available by the corresponding author upon reasonable request, subject to ethics approval, institutional data‐governance requirements and applicable data‐protection legislation.
